# A retrospective pilot study of transarterial chemoembolisation using camrelizumab-eluting Callisphere beads for unresectable hepatocellular carcinoma

**DOI:** 10.1186/s12885-023-11668-7

**Published:** 2023-11-24

**Authors:** Xiaonan Shi, Yang Wang, Jianzhuang Ren, Xinwei Han, Yonghua Bi

**Affiliations:** 1 Department of Oncology, the First Affiliated Hospital of Zhengzhou University, Zhengzhou, Zhengzhou China; 2 Department of Interventional Radiology, the First Affiliated Hospital of Zhengzhou University, Zhengzhou, China

**Keywords:** Camrelizumab, Transarterial chemoembolisation (TACE), Drug eluting transarterial chemoembolisation (DEB-TACE), Progression-free survival (PFS), Adverse events (AEs)

## Abstract

**Background:**

Our objective was to assess the efficacy and safety of initial hepatic arterial infusion of chemotherapy combined with transarterial chemoembolisation using camrelizumab-eluting Callisphere beads (camrelizumab-DEB-TACE) for treating unresectable hepatocellular carcinoma (HCC).

**Methods:**

Enrolment included patients with unresectable HCC who underwent camrelizumab-DEB-TACE treatment from September 2021 to February 2023. The assessment included the examination of tumour response, overall survival (OS), progression-free survival (PFS), and the monitoring of adverse events (AEs).

**Results:**

Twenty-one patients were included in the study. The objective response rates (ORR) and disease control rates (DCR) were 55.0% and 90.0% at 1 month and 57.9% and 78.9% at 3 months, respectively. The median PFS and OS were 7.4 and 15.5 months months, respectively. Among the 21 patients, 4 underwent more than 2 procedures of camrelizumab-DEB-TACE, with a mean of 1.9 ± 1.1 procedures (range: 1–4) per patient. No severe complications or treatment-related mortalities were observed. In addition, no patient developed severe AEs related to camrelizumab, such as reactive cutaneous capillary endothelial proliferation, immune-related pneumonia, or immune-related myocarditis. Nineteen patients experienced at least one type of AEs related to DEB-TACE, with abdominal pain (n = 16, 76.2%) being the most prevalent AE.

**Conclusion:**

Camrelizumab-DEB-TACE demonstrated effectiveness and safety as a treatment for unresectable HCC, with no occurrence of severe camrelizumab-related AEs.

## Background

Hepatocellular carcinoma (HCC) ranks among the most prevalent malignant tumours globally [1]. Given that the majority of patients receive a diagnosis at the intermediate or advanced stages, only a limited proportion (< 33.3%) can avail themselves of the benefits associated with surgical hepatectomy or liver transplantation [2, 3]. As an effective palliative therapy, both transarterial chemoembolisation (TACE) and drug-eluting beads TACE (DEB-TACE) are commonly employed in the management of unresectable, recurrent HCC, [4, 5] or residual intrahepatic HCC [6].

Combination therapy is pivotal in enhancing the efficacy of HCC. Studies indicate that TACE may stimulate the response of tumour-specific CD8 + T cells by releasing tumour-associated antigens [7]. Immune checkpoint inhibitors have been integrated into the therapeutic landscape for unresectable HCC [8–10]. Camrelizumab, an anti-programmed cell death protein 1 monoclonal antibody, has received approval in China to treat advanced HCC, demonstrating comparable efficacy with nivolumab and pembrolizumab [11]. However, when administered intravenously, camrelizumab exhibits a notable rate of treatment-related adverse events (AEs) (41.5%), notably the most common AEs such as reactive cutaneous capillary endothelial proliferation (RCCEP) (34.1%) [12]. In addition, DEB-TACE offer the advantage of sustained release of chemotherapy drugs and theoretically presents improved efficacy and safety compared to conventional TACE [13]. Hence, we hypothesised that DEB-TACE using camrelizumab-eluting Callisphere beads (camrelizumab-DEB-TACE) might be efficacious in treating HCC with enhanced safety. To our knowledge, no studies have reported on the clinical outcomes of camrelizumab-DEB-TACE in treating unresectable HCC. This study aims to present the safety and efficacy findings of camrelizumab-DEB-TACE in treating patients with unresectable HCC.

## Methods

### Patients

**Fig. 1:**
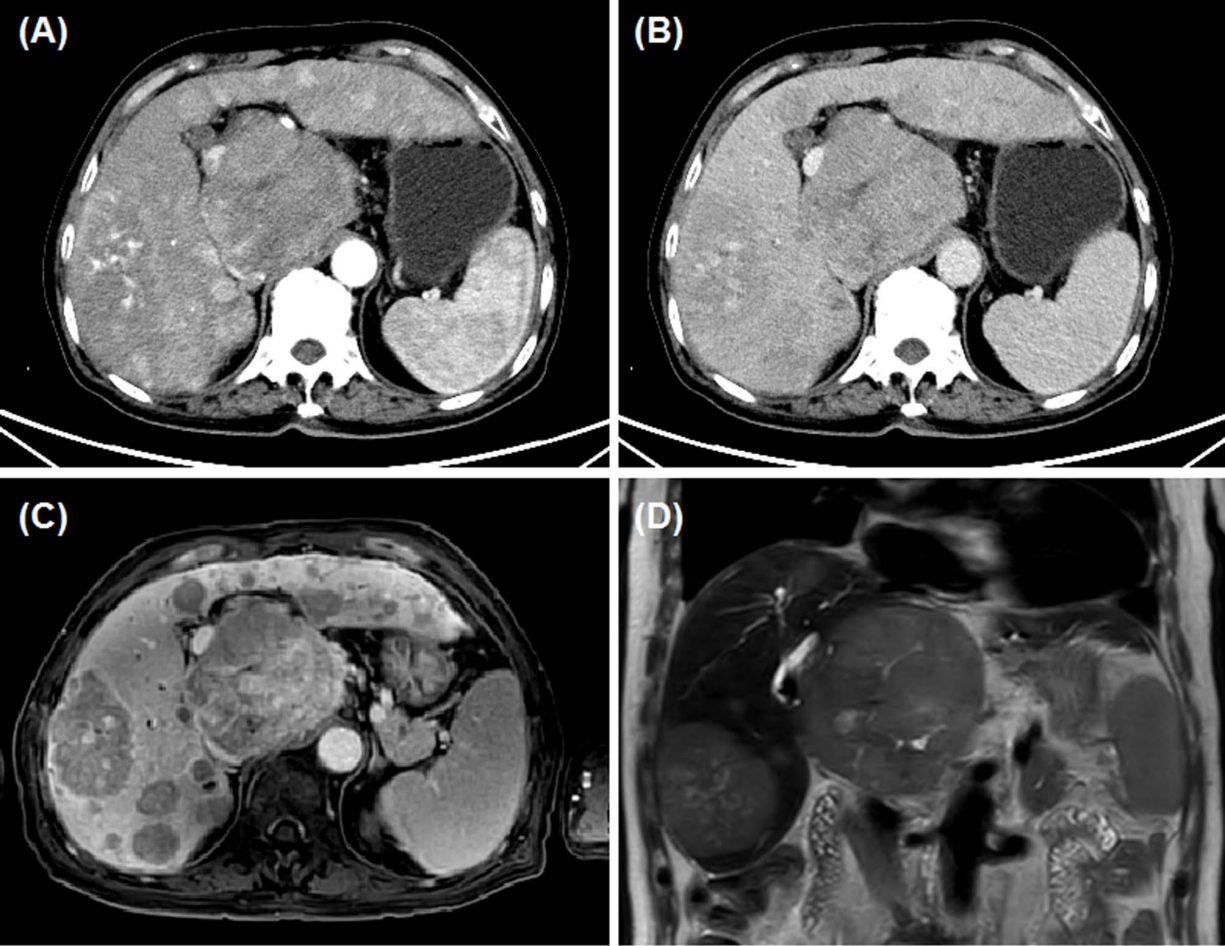
Abdominal contrast-enhanced CT and MRI for advanced primary HCC. **(A-B)** CT examination on admission revealed huge HCC with a maximum diameter of 94 mm, no portal vein invasion and multiple intrahepatic metastases. **(C-D)** MRI examination on admission revealed multiple cancer lesions in the left and right of liver lobes

**Fig. 2:**
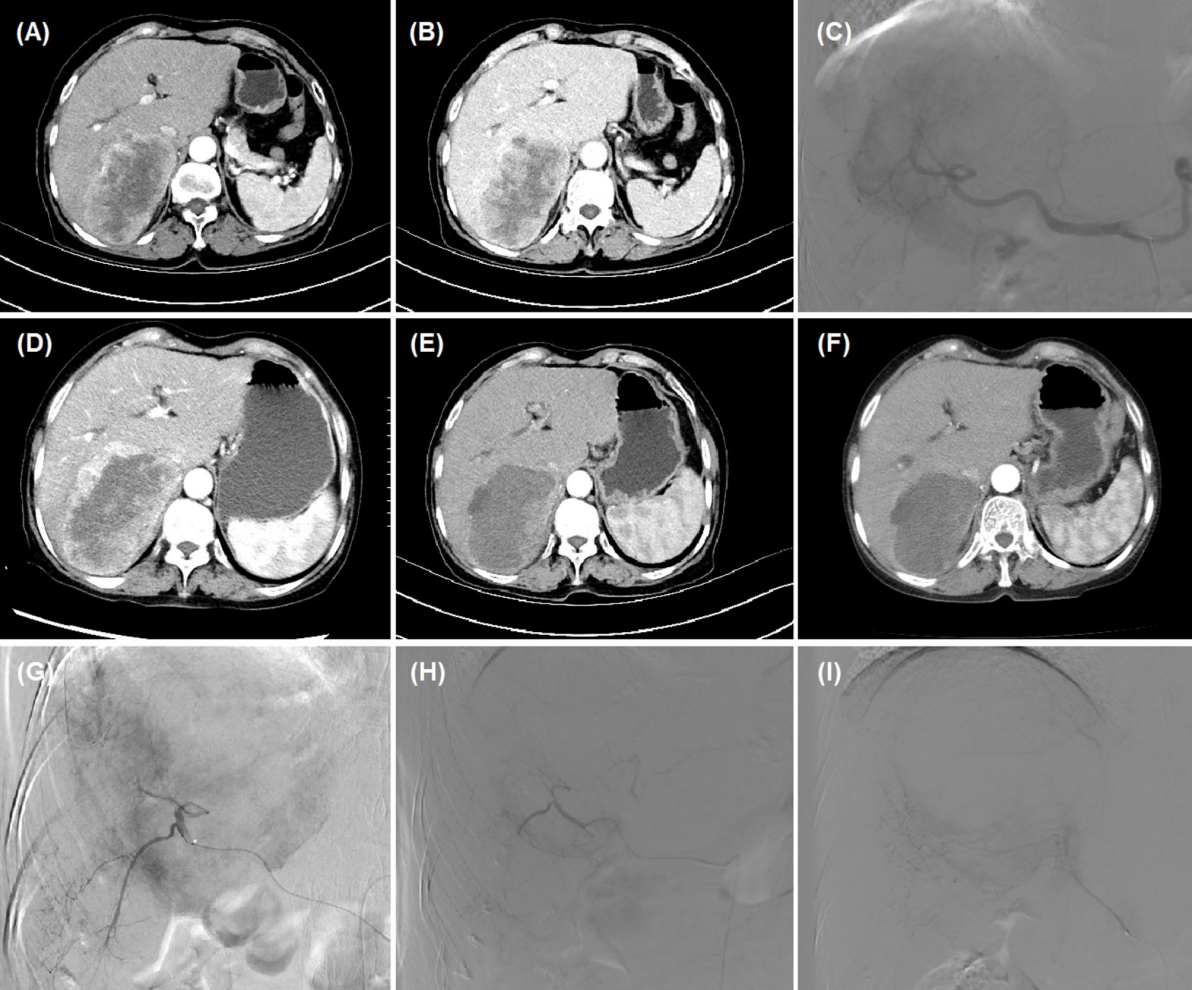
A female patient with for advanced primary HCC was treated by camrelizumab-DEB-TACE. **(A-B)** CT revealed a huge HCC in the right lobe. **(C)** A huge tumour staining was shown and the right hepatic artery was its blood supply artery. **(D, G)** The second DEB-TACE was conducted 1.7 months later for the residual tumour. **(E,H)** The third DEB-TACE was conducted 2.7 months after initial procedure. **(F,I)** The tumour was found to shrink after 4.1 months’ follow-up, and DEB-TACE was performed and right diaphragmatic artery was embolised by camrelizumab loading beads

This retrospective analysis included all consecutive patients with unresectable HCC who received camrelizumab-DEB-TACE between September 2021 and February 2023. The criteria for patient selection comprised individuals who met the following conditions: (1) age > 18 years; (2) patients with unresectable HCC or recurrent HCC after surgery or Barcelona Clinic Liver Cancer (BCLC) stage B or C (Figs. 1 and 2AB); (3) patients who received camrelizumab-DEB-TACE treatment; (4) Child–Pugh class A or B; and (5) an Eastern Cooperative Oncology Group (ECOG) performance status of 0–1. Exclusion criteria were defined as follows: (1) over 70% of the liver volume occupied by tumour tissue; (2) severe comorbidities; (3) allergy to study drugs.

Four patients had previously undergone conventional TACE unrelated to camrelizumab-DEB-TACE. Two patients underwent embolisation of gastric varices owing to previous cirrhosis hematemesis. Targeted therapy was co-administered with camrelizumab-DEB-TACE in 15 patients and thermal ablation was performed in seven patients. All patient details have been de-identified. The Institutional Review Board of the First Affiliated Hospital of Zhengzhou University approved all procedures conducted in this study. Informed consent was waived by the Institutional Review Board owing to the retrospective study design, and clinical data were retrospectively and anonymously analysed.

### DEB-TACE procedure

**Fig. 3:**
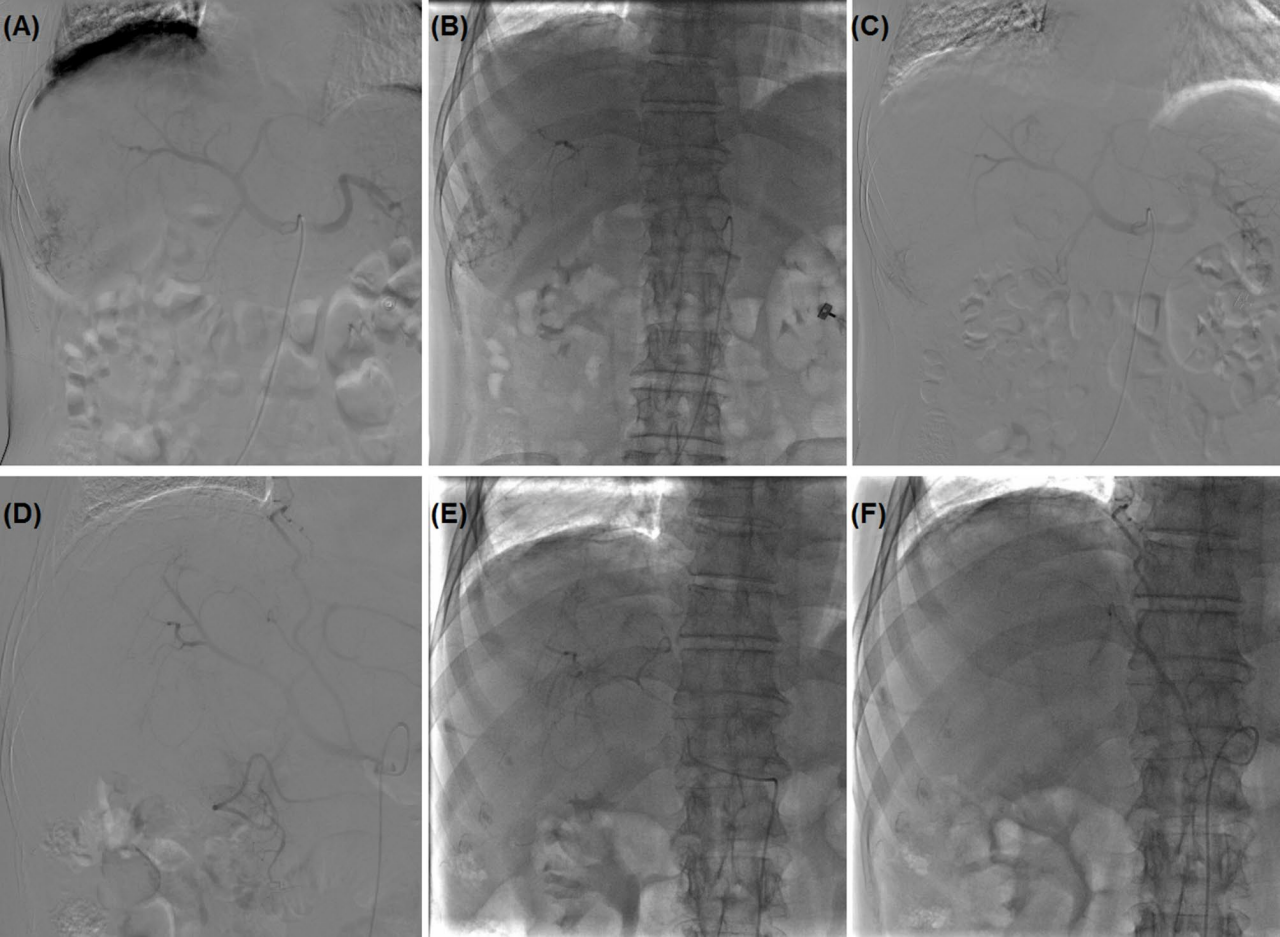
A male patient with advanced primary HCC was treated by camrelizumab-DEB-TACE. **(A)** A 5 F RH catheter was introduced for angiography to show the tumour and tumour-feeding arteries. **(B-C)** After super-selective catheterization of tumour-feeding arteries, 300–500 μm of drug eluting CalliSphere beads (loaded with 200 mg camrelizumab) were slowly injected until flow stasis. **(D)** The second DEB-TACE procedure was conducted about 1.5 months later for residual tumour mass. **(E-F)** The right hepatic artery and right diaphragmatic artery were embolised by camrelizumab loading beads

DEB-TACE was conducted under local anaesthesia and fluoroscopic guidance [14]. Seldinger’s method was employed to puncture the femoral artery, and a 5 F RH catheter was introduced for angiography to visualise the tumour and its feeding arteries. Following super-selective catheterisation of tumour-feeding arteries using a coaxial microcatheter (Progreat, Terumo), a preliminary hepatic arterial infusion of multiple chemotherapeutic agents (oxaliplatin 50–100 mg; doxorubicin 20–60 mg was subsequently performed, with or without raltitrexed 2–4 g). Subsequently, 100–300 or 300–500 μm drug-eluting CalliSphere beads (loaded with 200 mg camrelizumab, Hengrui Biomedical Technology Co., Ltd., Suzhou, China) were slowly injected until flow stasis was achieved. In cases of incomplete embolisation, 350–560 μm polyvinyl alcohol (PVA) particles (Hangzhou Alikang Pharmaceutical Technology Co., Ltd., Zhejiang, China) were used. The DEB-TACE procedure was repeated when a residual tumour mass was evident in enhanced computerised tomography (CT) or magnetic resonance imaging (MRI) during follow-up (Figs. 3 and 2C and G–I).

### Follow and evaluation

**Fig. 4:**
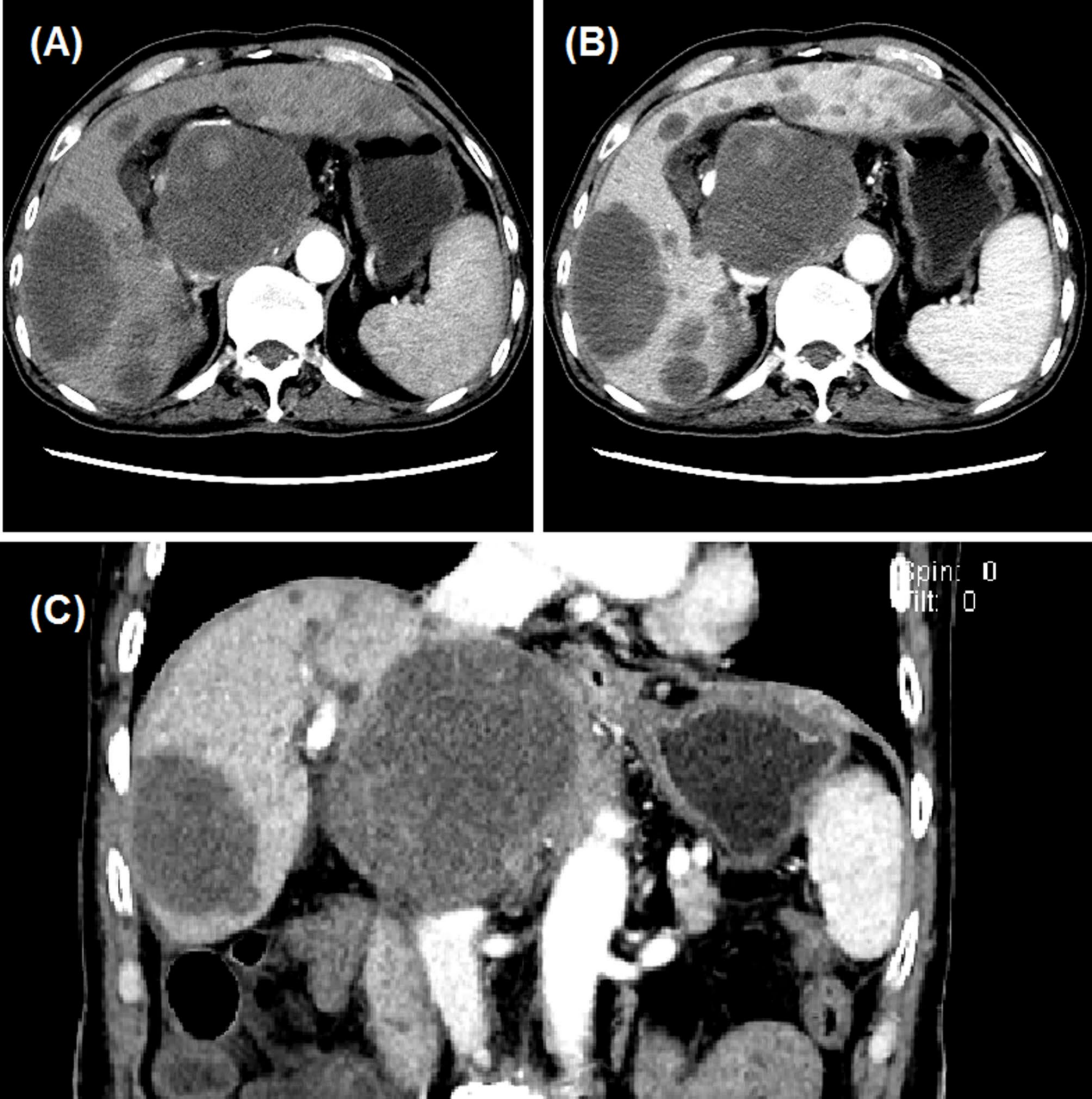
CT imaging follow up for the male patient with advanced primary HCC. **(A-C)** After 1.5 months’ follow-up, the tumour lesions were found to shrink, with almost no enhancement. PR was assessed according to mRECIST.

All patients were monitored until April 2023. Abdominal contrast-enhanced CT and/or MRI were conducted every 4 to 6 weeks following the initial DEB-TACE treatment (Figs. 4 and 2D–F). Follow-up CT and/or MRI at 1 and 3 months after the initial DEB-TACE were assessed in accordance with the Modified Response Evaluation Criteria in Solid Tumours (mRECIST). [15] The primary endpoints were progression-free survival (PFS), defined as the duration from the initial DEB-TACE to the date of disease progression, death or the last follow-up. AEs were evaluated using the Common Terminology Criteria for Adverse Events (CTCAE) Version 3.0 [16] and constituted the secondary endpoints.

### Statistical analyses

All analyses were conducted using Prism 5.0 software (GraphPad Software, Inc., San Diego, CA). Discrete variables were expressed as numbers with percentages, while quantitative data were presented as mean ± standard deviation. PFS and overall survival (OS) were calculated using the Kaplan–Meier method. A P-value < 0.05 indicated statistical significance.

## Results

### Patient characteristics

Between September 2021 and February 2023, 21 patients with HCC underwent camrelizumab-DEB-TACE treatment at our department. Among these patients, four individuals received more than two DEB-TACE procedures, with a mean of 1.9 ± 1.1 procedures (range: 1–4) per patient. The overall characteristics of the patients are detailed in Table 1. Notably, eight patients (36.6%) succumbed during the observation period of the study. A total of 39 DEB-TACE sessions were conducted, revealing extrahepatic feeders in eight procedures, including six right diaphragm arteries and two left gastric arteries. In addition, 30 DEB-TACE sessions were performed in the branches of the right hepatic arteries, four in the branches of the left hepatic arteries, and five in the bilateral hepatic arteries. The average inpatient duration was 10.1 ± 4.2 days, and the mean hospitalisation cost was (6.2 ± 1.8)×10^4^ ¥.

**Table 1:** Patient characteristics at admission

Parameters	Data
Sex, male, n (%)	16 (76.2%)
Mean age, years	62.5 ± 9.5
ECOG performance, n (%)	
0	11 (52.4%)
1	10 (47.6%)
Lesion distribution, n (%)	
Right lobe	12 (57.1%)
Left lobe	2 (9.5%)
Bilobar	7 (33.3%)
Symptom duration, months	1.3 (0.5, 4.5)
Co-administered targeted therapy	15 (71.4%)
Hepatitis virus infection, n (%)	
Hepatitis B virus	8 (38.1%)
Hepatitis C virus	2 (9.5%)
BCLC stage, n (%)	
B	5 (23.8%)
C	16 (76.2%)
Child-Pugh class, n (%)	
A	9 (42.9%)
B	12 (57.1%)
Portal vein invasion, n (%)	8 (38.1%)
Hepatic vein invasion, n (%)	7 (33.3%)
Extrhepatic metastasis, n (%)	8 (38.1%)
Lung	4 (19.0%)
Lymph node	2 (9.5%)
Bone	1 (4.8%)
Adrenal gland	1 (4.8%)
Tumour size, mm	52.2 ± 26.2
Ascites, n (%)	8 (38.1%)
Lesion number, n (%)	
≤ 3	18 (85.7%)
> 3	3 (14.3%)
a-Fetoprotein level, n (%)	16 (76.2%)
< 400 ng/mL	10 (47.6%)
≥ 400 ng/mL	6 (28.6%)

### Tumour Response

According to mRECIST, imaging assessments conducted 1 month after camrelizumab-DEB-TACE revealed that two patients (10.0%) achieved a complete response (CR), nine patients (45.0%) achieved a partial response (PR), and seven patients (35.0%) achieved stable disease (SD). Consequently, the overall response rate (ORR) and disease control rate (DCR) was 55.0% and 90.0%, respectively. Imaging results 3 months after camrelizumab-DEB-TACE demonstrated CR in three patients (15.8%), PR in eight patients (42.1%), and SD in four patients (21.1%). Therefore, the ORR and DCR were 57.9% and 78.9%, respectively (Table 2).

**Table 2:** Local tumour response assessed using the mRECIS criteria

Response	1 month	3 months
Complete response	2 (10.0%)	3 (15.8%)
Partial response	9 (45.0%)	8 (42.1%)
Stable disease	7 (35.0%)	4 (21.1%)
Progressive disease	0 (0.0%)	1 (5.3%)
Not evaluable	2 (10.0%)	3 (15.8%)
Overall response rate	11 (55.0%)	11 (57.9%)
Disease control rate	18 (90.0%)	15 (78.9%)

### Survival outcomes

**Fig. 5:**
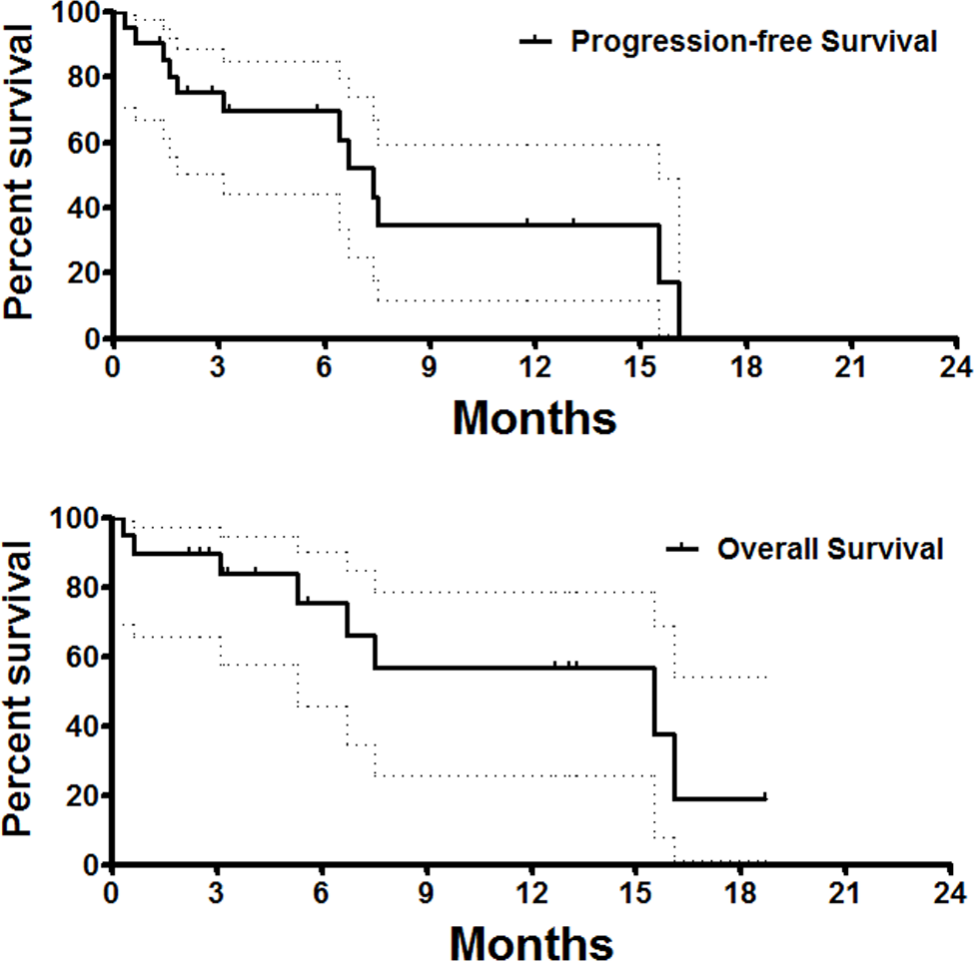
The OS and PFS follow-up. The 3-, 6-, and 12-month OS rates were 90.0%, 75.6% and 56.7%, respectively. The 3-, 6-, and 12-month PFS rates were 75.4%, 69.6% and 34.8%, respectively

One patient was lost to follow-up, and the median follow-up period was 12.7 months. Among the 21 patients, 12 (57.1%) developed progressive disease (PD) during follow-up. The median PFS and OS were 7.4 and 15.5 months months, respectively. The 3-, 6-, and 12-month OS rates were 90.0%, 75.6%, and 56.7%, respectively. The 3-, 6-, and 12-month PFS rates were 75.4%, 69.6%, and 34.8%, respectively (Fig. 5).

### AEs

A total of 19 patients experienced at least one type of postembolisation syndrome, including fever (n = 6), abdominal pain (n = 16), and nausea and/or vomiting (n = 8) within 5 days after the procedure. AEs related to DEB-TACE, with abdominal pain (n = 16, 76.2%), constituted the most common AE. All symptoms were alleviated or improved following symptomatic treatment. Furthermore, no severe complications or treatment-related mortalities were reported.

Throughout the follow-up period, no patients developed severe AEs related to camrelizumab, such as RCCEP, immune-related pneumonia, or immune-related myocarditis (Table 3).

**Table 3:** Clinical data on DEB-TACE

Variables	Data
Inpatient duration, days	10.1 ± 4.2
Hospitalization cost, ×10^4^ ¥	6.2 ± 1.8
DEB-TACE procedures	
Mean	1.9 ± 1.1
≤ 2 procedures, n (%)	17 (81.0%)
> 2 procedures, n (%)	4 (19.0%)
Other related interventional treatments	
Co-administered thermal ablation	7 (33.3%)
Conventional TACE prior to DEB-TACE	4 (19.0%)
Previous embolisation of gastric varices	2 (9.5%)
DEB-TACE-related AEs, n (%)	19 (90.5%)
Fever	6 (28.6%)
Nausea and/or vomiting	8 (38.1%)
Liver abscess	1 (4.8%)
Thrombocytopenia	1 (4.8%)
hyperbilirubinemia	2 (9.5%)
Leukopenia	2 (9.5%)
Abdominal pain	16 (76.2%)
Raised ALT/AST	5 (23.8%)
Camrelizumab-related AEs, n (%)	0 (0.0%)
RCCEP	0 (0.0%)
Immune-related pneumonia	0 (0.0%)
Immune-related myocarditis	0 (0.0%)

DEB-TACE = drug eluting beads transarterial chemoembolization; ALT = Alanine aminotransferase; AST = Aspertate aminotransferase; TACE = Transcatheter arterial chemoembolization; RCCEP reactive cutaneous capillary endothelial proliferation

## Discussion

The advent of immune checkpoint inhibitors has propelled advancements in HCC treatment. The HCC tumour microenvironment is characterised by the infiltration of cells such as natural killer cells and T cells, and so on [17]. Bland hepatic artery embolisation induces changes in the peripheral blood T-cell environment, leading to reductions in regulatory T cells and type 1 helper T cells, thus providing a rationale for combined immunotherapy with TACE [18].

TACE is recommended as the first-line therapy for intermediate BCLC B HCC, and the China Liver Cancer Staging (CNLC) model expands its indications [19]. According to Chinese clinical guidelines on HCC management, TACE is indicated for CNLC stage IIb (≥ 4 nodules, Child-Pugh A/B, PS 0–2), IIIa (Vascular invasion, Child-Pugh A/B, PS 0–2) and some IIIb (Metastases, Child-Pugh A/B, PS 0–2) patients with HCC [20]. TACE induces tumour necrosis in HCC and elicits the release of tumour-associated antigens, promoting a tumour-specific CD8 + T cell response [7]. Zhan et al. [9] reported the safety of radioembolisation combined with checkpoint inhibitor immunotherapy in HCC patients, with limited treatment-related toxicity. Marinelli et al. [10] demonstrated the efficacy of transarterial radioembolisation or chemoembolisation combined with nivolumab immunotherapy in patients with intermediate and advanced HCC. Camrelizumab has gained approval in China for treating advanced HCC, exhibiting comparable efficacy with nivolumab and pembrolizumab. [11] Furthermore, camrelizumab has shown effectiveness in patients experiencing untreatable progression of HCC after initial TACE treatment [12].

DEB-TACE theoretically holds the potential for improved outcomes compared to conventional TACE, and diverse loading drugs have been employed in patients with advanced HCC [4, 14, 21]. The pH of the carelizumab is 5.0–5.8, rendering it positively charged in vitro. The isoelectric point of the antibody is 7.9, exceeding the body’s pH range (7.35–7.45), thus maintaining a positive charge in the body. CalliSphere beads with negative charges can form an absorptive interaction with the positively charged carelizumab. In addition, CalliSphere beads loaded with various drugs have been extensively used in patients with advanced HCC [4, 14, 21]. Consequently, in theory, DEB-TACE using camrelizumab eluting Callisphere beads (camrelizumab-DEB-TACE) may effectively treat HCC with enhanced safety. No studies have reported the clinical outcomes of camrelizumab-DEB-TACE in the treatment of unresectable HCC. In this study, camrelizumab-DEB-TACE treatment achieved a median PFS of 7.4 months in unresectable HCC patients, surpassing the reported duration for systemic camrelizumab therapy in patients with HCC [12].

Regarding tumour response, camrelizumab-DEB-TACE demonstrated comparable outcomes for unresectable HCC. Previous studies have reported an ORR of 14.7% after camrelizumab treatment in patients with advanced HCC who had failed previous sorafenib or chemotherapy therapy [11]. The ORR and DCR were 12.2% and 58.5%, respectively, at 6 months after camrelizumab therapy in patients with HCC with TACE failure [12]. In the current study, the ORR and DCR were 57.9% and 78.9%, respectively, at 3 months after DEB-TACE.

RCCEP is more frequently observed in patients undergoing camrelizumab treatment [22]. The reported incidence of RCCEP was 67.0% in advanced HCC [11], and it has been documented at 76.7% after camrelizumab monotherapy in advanced oesophageal carcinoma [23]. Considering that AEs, such as fatigue and diarrhoea, attributed to camrelizumab can potentially be toxicities of all other co-administered interventions, distinguishing camrelizumab AEs from other AEs is challenging. Furthermore, non-characteristic, minor AEs are subjective sensations that are not easily objectively described and quantified in this retrospective study. Therefore, our focus was solely on AEs that were relatively specific and severe. Notably, no patient experienced severe AEs related to camrelizumab, such as RCCEP, immune-related pneumonia, or immune-related myocarditis. This study revealed that 90.5% of patients had AEs related to the DEB-TACE procedure, with abdominal pain being the most common. Patients developed at least one type of postembolisation syndrome, such as fever, abdominal pain, nausea and/or vomiting within 5 days after the procedure.

Doxorubicin-loading beads are primarily used in standard DEB-TACE for patients with unresectable HCC [24–27]. Currently, camrelizumab-DEB-TACE is not the standard TACE, and this preliminary study was conducted to investigate its safety and efficacy in treating unresectable HCC patients. According to the instructions for use and published studies, camrelizumab was administered at a dose of 200 mg (for body weight ≥ 50 kg) or 3 mg/kg (for body weight < 50 kg) for patients with advanced HCC [12, 28]. Thus, in this study, 200 mg camrelizumab was loaded with CalliSphere beads. Hepatic arterial infusion chemotherapy has been indicated for patients with major portal vascular invasion or with Child-Pugh B liver function [20]. In real-life practices, preliminary hepatic arterial infusion of multiple chemotherapeutic agents was performed before DEB-TACE to enhance clinical outcomes. Such hepatic arterial infusion chemotherapy was also performed before DEB-TACE in previous studies involving patients with HCC [4, 14, 21]. Additionally, patients who would have preferably been treated with systemic therapy also received systemic therapy, such as targeted therapy, in cases of extrahepatic metastasis.

Several limitations should be acknowledged in this study. First, being a retrospective study conducted in a single institution, the sample size is small. Second, the period covered is relatively short, warranting investigation into long-term outcomes. Third, the study lacks a control group to confirm the superiority of camrelizumab-DEB-TACE over camrelizumab monotherapy. In addition, a phase I clinical study was not conducted, resulting in missing information on drug-eluting time and pharmacokinetics of camrelizumab. Notably, the therapy described in this study involves combined hepatic artery infusion of chemotherapy plus camrelizumab-DEB-TACE, and as such, all response and survival statistics reflect the impact of these combined therapies and not camrelizumab alone. Therefore, a multicentre prospective randomized trial with comparative studies is needed to confirm the clinical outcomes of camrelizumab-DEB-TACE in unresectable HCC.

In conclusion, this study represents the first report of camrelizumab-DEB-TACE in the treatment of patients with unresectable HCC. The results indicate that camrelizumab-DEB-TACE offers an effective and safe treatment for unresectable HCC with no severe camrelizumab-related AEs.

## Data Availability

The datasets generated during and/or analyzed during the current study are available from the corresponding author on reasonable request.
